# Sphingosine-1-Phosphate Signaling in Cardiovascular Diseases

**DOI:** 10.3390/biom13050818

**Published:** 2023-05-11

**Authors:** Na Wang, Jing-Yi Li, Bo Zeng, Gui-Lan Chen

**Affiliations:** Key Laboratory of Medical Electrophysiology, Ministry of Education & Medical Electrophysiological Key Laboratory of Sichuan Province, Institute of Cardiovascular Research, Southwest Medical University, Luzhou 646000, China

**Keywords:** Sphingosine-1-phosphate, Sphingosine-1-phosphate receptor, cardiovascular disease, signaling mechanism

## Abstract

Sphingosine-1-phosphate (S1P) is an important sphingolipid molecule involved in regulating cardiovascular functions in physiological and pathological conditions by binding and activating the three G protein-coupled receptors (S1PR1, S1PR2, and S1PR3) expressed in endothelial and smooth muscle cells, as well as cardiomyocytes and fibroblasts. It exerts its actions through various downstream signaling pathways mediating cell proliferation, migration, differentiation, and apoptosis. S1P is essential for the development of the cardiovascular system, and abnormal S1P content in the circulation is involved in the pathogenesis of cardiovascular disorders. This article reviews the effects of S1P on cardiovascular function and signaling mechanisms in different cell types in the heart and blood vessels under diseased conditions. Finally, we look forward to more clinical findings with approved S1PR modulators and the development of S1P-based therapies for cardiovascular diseases.

## 1. Introduction

Sphingosine-1-phosphate (S1P) was discovered in the 1960s as a metabolic product of sphingolipids [1]. The biosynthetic reactions are mediated by a sequence of enzymes. In short, S1P is formed through the phosphorylation of sphingosine (Sph) catalyzed by Sph kinase 1 (SphK1) or Sph kinase 2 (SphK2). Afterwards, ceramide is broken down by ceramidases into sphingosine [2]. Then, several transporters and S1P receptors address S1P to the right position to perform its function. Degradation of S1P occurs through two pathways: the irreversible degradation of S1P to hexadecenal and phosphoethanolamine via S1P lyase (SGPL1), while reversible dephosphorylation of S1P to Sph bears by S1P phosphatases (SPP1 and SPP2) or broad-specificity lipid phosphate phosphatases (LPPs) [3]. It is well known that S1P is a potent bioactive sphingolipid and participates in signal transduction of a host of cellular processes such as cell growth, differentiation, proliferation, migration, and apoptosis [4]. S1P additionally serves as an intracellular secondary messenger in response to various extracellular stimuli. In this process, several intracellular targets, such as histone deacetylases (HDAC1 and HDAC2), TNF receptor associated factor 2 (TRAF-2), and the apoptosis inhibitor cIAP2, interact directly with S1P to influence a variety of cellular processes, including gene transcription and apoptosis [5,6].

Cardiovascular disease (CVD) is one of the leading causes of death in the world. The pathogenesis and treatment of CVD have always been a difficult and hot topics in cardiovascular research. Recently, abnormal levels of sphingolipid molecules have been detected in the plasma or blood vessels of patients with atherosclerosis, hypertension, and other CVD [7,8], indicating that sphingolipid molecules may play a crucial role in the occurrence and development of CVD. Accumulating evidence points to the engagement of S1P in inflammation, the vascular endothelial barrier, immune cell trafficking, stress response, angiogenesis, and the contraction and relaxation of smooth muscle [9,10,11]. Owing to the significance of S1P in cardiovascular physiology and pathophysiology, we reviewed the regulatory networks and functions of S1P and its receptors (S1PRs) in the cardiovascular system and the signaling mechanisms of S1P in various cardiovascular diseases.

## 2. Regulatory Network of S1P and S1PRs

**Figure 1:**
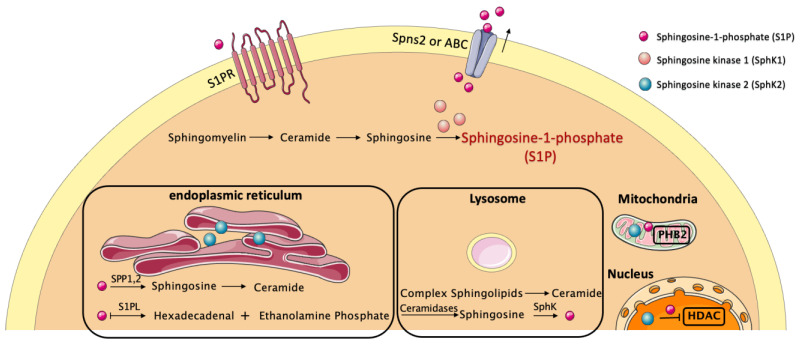
The biosynthesis, secretion and degradation of S1P. Sphingosine kinases 1 and 2 (SphK1 and SphK2) catalyze the phosphorylation of sphingosine to S1P after ceramide is broken down by ceramidases into sphingosine. SphK1 is mainly in the cytoplasm, and SphK2 is located within the mitochondria, nucleus, and endoplasmic reticulum (ER). In the mitochondria and nucleus, SphK2 also produces S1P as a second messenger with targets such as prohibitin 2 (PHB2) and histone deacetylases (HDACs). After being transported out of the cell by ATP-binding cassette (ABC) transporters and Spinster Homolog 2 (Spns2) protein, S1P acts predominantly through the binding and activation of different subtypes of S1PRs and initiates downstream signaling. S1P can be degraded in the ER through two pathways: dephosphorylation of S1P by S1P phosphatase (SPP1,2) to sphingosine, which can be used for ceramide synthesis, or irreversible degradation by S1P lyases (SPL).

Homeostatic S1P levels are maintained through their generation, transport, and degradation (Figure 1). SphKs modulate the balance between S1P, Sph, and ceramides in the synthesis process. Venkataraman et al. [12] showed that Sphk1^−/−^ mouse plasma has undetectable Sphk activity and that S1P levels were reduced by approximate 65%. They speculated that the Sphk1a isoform might contribute to the establishment of an S1P gradient in the vasculature. Nagahashi et al. [13] confirmed a “S1P gradient” in blood, lymphatic fluid, and peripheral lymphatic tissues. In contrast, they found that SphK2 knockout mice had significantly higher S1P levels in the lymphatic fluid than those in the wild-type mice. Overall, their results suggest that SphK1 and SphK2 have opposite effects on S1P concentrations. The expression of SphKs varies in different tissues and organs, with high levels of SphK1 present in the lung, spleen, and liver, and SphK2 predominantly present in the liver and in heart [14].

S1P is mainly derived from erythrocytes, endothelial cells (ECs), and platelets and maintains high concentrations in the blood and lower levels in other tissues [15]. S1P levels in human plasma range between 0.1 and 1.2 µM [16]. A component of high-density lipoprotein (HDL), apolipoprotein M (ApoM), binds S1P stably and plentifully, whereas the remaining amount of S1P is bound and transported via albumin (30–40%). Less than 10% of S1P is transported by low-density lipoproteins (LDLs) and very low-density lipoproteins (VLDLs) [17]. As S1P is known to be synthesized intracellularly, its export into the blood requires transporter molecules. It has been reported that spinster homolog 2 (Spns2) from vascular ECs facilitates S1P export into the blood, although some studies have suggested that Spns2 is required for the secretion of lymphatic but not plasmic S1P [18,19]. The other transporter, the major facilitator superfamily transporter 2b (Mfsd2b), is responsible for the export of S1P from erythrocytes and platelets [20]. In addition, the ATP-binding cassette (ABC) transporter family and ABCC4/MRP4 (multidrug resistance protein 4) are both involved in the secretion of S1P [21,22]. Furthermore, the removal of S1P is tightly regulated by eight S1P-metabolizing enzymes. Research shows that the cellular levels of sphingolipid metabolites are altered by an enforced expression of S1P phosphohydrolase in yeast and mammalian cells, increasing sphingosine and ceramide, which often have opposing biological effects to S1P [23]. Animal experiments indicated that oral dosing of the SPL inhibitor in rats caused increased cardiac S1P levels [24]. Treatment of S1P phosphohydrolase 1 (SPP-1)-overexpressing cells with S1P revealed that SPP-1 regulates ceramide levels in the endoplasmic reticulum (ER) [25]. LPP2 and LPP3 were reported to induce a reduction in the basal levels of S1P in HEK293 cells [26]. By modulating the ratio of ceramide/sphingosine and S1P, these S1P-metabolizing enzymes are considered key factors in determining cell survival or death [27]. 

As S1P acts predominantly through the activation of different subtypes of S1PRs, the spatial distribution of S1PRs is a major determinant of S1P’s versatile roles in physiological processes. It has been reported that S1PR1, 2, and 3 are ubiquitously expressed in all tissues and organs, including the cardiovascular system [3]. In contrast, the expression of S1PR4 and 5 is more tissue-specific, with S1PR4 expressed in the immune system and S1PR5 expressed in the immune and nervous systems. It is recognized that the S1P/S1PRs axis activates G proteins of different isotypes, initiating different downstream molecular signals, and widely participating in cell signal transduction and functional regulation [28]. There are four subclasses of the Gα subunit (Gα_i/o_, Gα_q_, Gα_12/13_, and Gα_s_). Gα_i/o_ extensively couples with S1PR1-5 and facilitates the activation of the small GTPases Ras and Rac, phosphatidylinositol-3-kinase (PI3K)/Akt, and phospholipase C (PLC) to promote cell proliferation, migration, apoptosis inhibition, and intracellular free calcium as well as inhibit adenylyl cyclase activity to reduce cAMP [28]. Gα_12/13_ and Gα_q_ couple with S1PR2/3/4/5 and S1PR2/3, respectively, which primarily activate the small GTPase Rho/ROCK and PLC pathways to inhibit migration [28]. Other studies have suggested that Gα_s_ act as relay mediators of S1PR1 signaling to control vascular integrity [29]. In addition, S1PR1/2/3 were reported to bind Gβγ subunits to activate the inwardly rectifying K^+^ current or the AKT and ERK pathways in cardiomyocytes [30,31]. However, the correlation and mechanism between them require further investigation. 

The cryo-electron microscopy (cryo-EM) structures of the S1PR1-Gα_i_ complex [32,33,34], S1PR2-Gα_13_ complex [35], S1PR3-S1P complex [36], and S1PR5-Gα_i_/inverse agonist [37,38] have been resolved recently. Structural analyses suggested that the S1PR family possesses a highly conserved long channel shape of the orthosteric site and revealed conformational changes associated with activation and differences in ligand access. A very similar receptor activation mechanism might be shared by multiple S1PRs, which is dependent on some key residues composing the orthosteric binding pockets [32]. These findings can potentially promote the design of G protein subtype-selective agonists to optimize receptor-targeting therapy and obtain S1PR structures that bind to different ligands in multiple conformations, which is critical for reducing the side effects of S1PR-targeting drug discovery. 

## 3. S1P and Cardiovascular Functions

**Figure 2:**
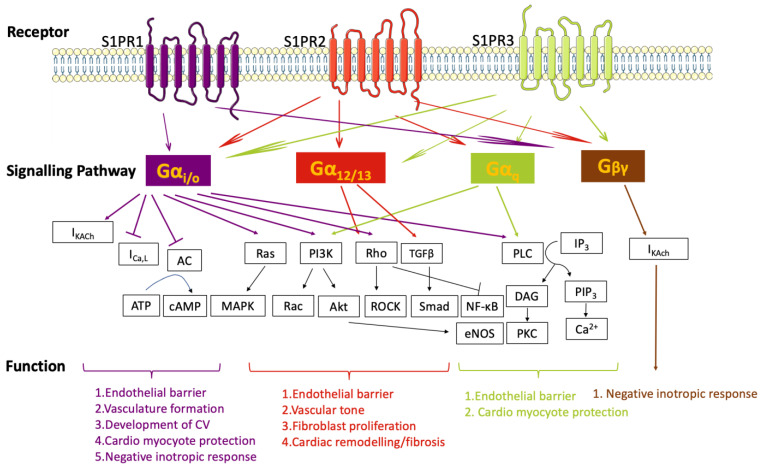
S1PRs-mediated signaling and physiological consequences. S1PR1, 2, and 3 are expressed in the cardiovascular system and play important roles in cell proliferation, migration, and apoptosis inhibition. S1PR-associated signaling pathways are involved in the regulation of endothelial barrier maintenance, vasculogenesis, vascular tone, inflammation, cardiac muscle contraction, heart rate, and fibrosis. The complex actions of S1P are dependent on the diversity of S1PRs coupled with distinct G protein subtypes.

The effects of S1P on the cardiovascular system occur mainly through S1PR1-3 and downstream signaling pathways (Figure 2). S1P transporters and S1P-metabolizing enzymes are also reported to affect the functions of S1P. 

### 3.1. Effects of S1P in Blood Vessels

In ECs, S1P plays an important role in endothelial barrier maintenance, vasculogenesis, vascular tone, inflammation, lymphocyte trafficking, chemotaxis, and immunity via its receptors S1PR1-3 [39,40]. The occurrence of these cellular processes depends on the concentration of S1P and differential patterns of S1PR expression within tissues, along with the diversity of G-proteins they couple [9]. In most ECs, S1PR1 is highly expressed, followed by S1PR3, and to a lesser extent S1PR2 [41]. In vivo and in vitro studies reported that S1PR1 protected or rescued endothelial barrier function in ECs and might act through Gα_i_ to activate the small GTPase Rac/Cdc42 or the PI3K/ERK pathway [40]. Interestingly, a study with S1PR1-GFP reporter mice found that S1PR1 activated EGR1 and STAT3 during EC injury, which transcribe SphK1 and Spns2 to promote S1P generation and efflux, committing vascular repair [42]. In contrast to S1PR1, S1PR2 was shown to activate the small GTPase Rho/ROCK and the phosphatase and tensin homologue (PTEN) pathway, and inhibit AKT phosphorylation to disrupt adhesion junctions and increase paracellular permeability [43]. Liu et al. [44] reported that S1PR2 expression increased in cultured human umbilical vein endothelial cells (HUVECs) in response to high glucose, and S1PR2 antagonist could activate PI3K/AKT/glycogen synthetic kinase 3β (GSK-3β), signaling pathway and inhibit NO production against high glucose-induced mitochondrial apoptosis and ECs damage. S1PR3 has been shown to mediate cell proliferation and vascular permeability and has been proposed as a potential biomarker in acute lung injury (ALI) due to its elevated total plasma concentrations (>251 pg/mL) in ALI [45]. 

The endothelial glycocalyx (EG) is a jelly like protective layer covering the luminal surface of the endothelium and forms part of the barrier [46]. Plenty of research has demonstrated that S1P is involved in its synthesis through PI3K signaling and stabilizes its structure by suppressing the activity of metalloproteinases, which mediate EG shedding or restore interendothelial transport by preventing EG shedding via gap junctions [46,47,48,49]. S1P was also confirmed to improve endothelial barrier integrity in vivo, prevent excess microvascular permeability in rats, and exhibit protective effects in patients after hemorrhagic shock [50,51]. In addition, in the absence of S1PR1 in ECs, impaired inhibition of vascular endothelial growth factor receptor 2 (VEGFR2) leads to disruption of vascular barrier function and induces aberrant angiogenesis [52]. However, S1PR1 supports VEGFR2-mediated angiogenesis through Gα_i_ signaling during tumor growth [53]. Moreover, S1PR3-dependent upregulation and activation of the VEGFR2 pathway was suggested to be the mechanism underlying the proangiogenic effects of HDL [54]. Wang et al. [55] found the S1P-rich microenvironment supported by Mfsd2a and Spns2 in the extracellular matrix (ECM) of vascular endothelium dominates the formation and maintenance of the blood-brain barrier. Recently, lung ECs isolated from Spns2 deficient mice revealed increased leakage of dextran and decreased resistance in electric cell-substrate impedance sensing (ECIS) measurements, indicating that secretion of S1P by ECs via Spns2 contributed to constitutive EC barrier maintenance [56]. Studies have investigated the role of S1P in regulating vascular tone and confirmed that S1P/S1PR1/3 signaling in ECs promotes endothelium-dependent vasorelaxation by activating of AKT/endothelial nitric oxide synthase (eNOS) and NO production [57]. 

In vascular smooth muscle cells (VSMCs), it has been reported that elevated S1PR1 expression in response to angiotensin II (Ang II), via H_2_O_2_-mediated APE/Ref1 translocation, induces VSMCs migration and vascular neointima formation [58]. Kerage et al. [59] used eNOS KO mice and found that S1P/S1PR2/3 signaling in VSMCs mediated vasoconstriction and anti-inflammation through PI3K/AKT/eNOS signaling pathway reduce NO. Emerging evidence from the past decades indicates that S1P/S1PR1-3 signaling mainly regulate VSMC proliferation, migration, and VSMC-dependent vascular tone [60]. However, it was also shown that S1P-induces the release of tissue inhibitors of metalloproteinase-2 (TIMP-2) via the activation of Rho kinases in VSMCs inhibited angiogenesis [61]. 

Circulating S1P could also produce natriuretic effects by acting on epithelial sodium channels via activation of S1PR1 in the renal medulla, causing significant increase in urine flow and sodium excretion [62], and it could also regulates blood pressure through effects on blood volume. It is worth noting that the immunomodulatory drug fingolimod (FTY720) approved for oral treatment of relapsing-remitting multiple sclerosis [63], increases blood pressure by significant downregulation of vascular endothelial S1PR1 [64]. Although FTY720 is a potent agonist of S1PR1/3/4/5, its application consequently causes the internalization and degradation of S1PRs and is thus considered a functional antagonist of S1PRs [65]. Based on the study of these two types of cells, it is reasonable to presume that intervention in vascular ceramide accumulation or regulation of the S1PR1 signaling pathway may be an effective strategy for antihypertensive drugs.

### 3.2. Effects of S1P in the Heart

It has been established that S1P affects cardiac muscle contraction, heart rate, and cardiac fibroblasts. S1PR1-3 are the main isoforms of S1PRs expressed in the heart, and the expression patterns of these receptor subtypes in different cardiac cell types directly reflect differential regulatory effects and cardiac performance [66]. It is recognized that S1PR1 is predominantly expressed in cardiomyocytes, S1PR3 is the most common subtype in fibroblasts, and S1PR2 is expressed in both of them. S1P-S1PR1 signaling resulted in decreased myocyte shortening and negative inotropic effects by coupling Gα_i_ to reduce cAMP accumulation-associated decrease in L-type calcium channel current, or coupling Gβγ to activate inwardly rectifying K^+^ current and shortening action potential duration (APD) [30]. Another study showed that the S1P-S1PR2/3-Gα_i_ pathway activates AKT and ERK in adult mouse ventricular myocytes to inhibit isoproterenol-stimulated cAMP accumulation, leading to a negative inotropic response [31]. Cardiomyocyte-specific S1PR1 knockout mice displayed progressive cardiomyopathy, compromised response to dobutamine, and premature death, which further confirmed the crucial role of S1P signaling in mediating cardiac muscle contraction [67].

S1P also inhibits heart rate, mainly by activating S1PR1. Several studies have reported that long-term treatment with S1PR modulators could reduce cardiac autonomic activation and decrease heart rate variability by binding S1PRs to trigger G protein-gated inwardly rectifying potassium (GIRK) channels on cardiac myocytes [68,69]. Atrial GIRK channels are activated via Gβγ to induce a transient decrease in heart rate. S1P also regulates the hyperpolarization-activated inward current (*I*_f_) and the voltage-gated calcium current (*I*_L-Ca_) in rabbit sinoatrial node cells and mediates the decrease in heart rate only in the presence of the beta-adrenergic agonist isoproterenol [70]. Therefore, S1P is considered a potential target for arrhythmia due to its important role in regulating heart rhythm.

## 4. S1P and Cardiovascular Diseases

### 4.1. Anti-Atherosclerotic Effect of S1P

Atherosclerosis (AS) is characterized by the focal accumulation of lipids in arterial walls and is identified as the effect of hemodynamic wall shear stress (WSS) on endothelial permeability. In recent years, it has been found that S1P is associated with HDL particles and produces antiatherosclerotic effects [71,72,73]. Sphk1 inhibitors significantly reduced plasma S1P levels and aggravated atherosclerotic lesions in low-density lipoprotein receptor (LDLR) knockout mice [74], while the deficiency of S1P lyase alleviated atherosclerotic lesions [75]. Patients with coronary artery disease (CAD) showed reduced S1P content in HDL compared to healthy individuals [76]. In CAD, S1P-loading improves HDL dysfunction in vitro and in vivo [77]. Additionally, therapeutic S1P-loading boosted the anti-inflammatory function of HDL in VSMCs via S1PR2 but not S1PR1 or S1PR3 [72]. The aforementioned S1PR agonist FTY720 and its two derivatives (ST-968 and ST-1071) have been shown to reduce the expression of adhesion molecules of immune cells by activating the S1PR3-PI3K/AKT signaling pathway and exert antiatherosclerotic effects [78]. Therefore, the S1P signaling pathway may serve as a potential drug target for the prevention and control of atherosclerosis. However, S1P was also shown to promote thrombosis by acting on lymphocytes, breaking the traditional notion that S1P is only protective against AS [79]. A recent summary of clinical findings suggests that S1P bound in HDL3-apoM complexes produces antiatherogenic effects by activating S1PR1 in endothelial cells, thus exhibiting an overall vasculoprotective effect [80].

### 4.2. S1P Promotes Angiogenesis

Angiogenesis refers to the process of endothelial cell proliferation and migration, which depends on pro- and anti-angiogenic molecules. Well-regulated angiogenesis plays a pivotal role in many physiological conditions such as embryonic development, wound healing, and endometrial changes in the menstrual cycle. Angiogenesis is also the basis of various pathological processes, including tumor metastasis and atherosclerotic plaque formation [81]. It has been reported that S1P promotes angiogenesis through S1PR/VEGFR to regulate endothelial cell proliferation and migration [54,82]. S1P and downstream signaling pathways in pathological angiogenesis are promising targets for disease treatment. SphK inhibitors, S1P monoclonal antibodies, and S1PR modulators are currently the main pharmacological agents targeting the S1P axis. For example, FTY720 has been reported to inhibit angiogenesis by targeting S1PR and VEGF-induced migration of vascular endothelial cells [83]; JTE013, a selective antagonist for S1PR2, improved postischemic angiogenesis via the AKT/eNOS signaling pathway [84]. Recently, Wilkerson and his colleagues established an in vivo model of corneal neovascularization to study the role of S1P in angiogenesis [85], which will be very useful for monitoring the process of neovascularization and angiogenesis in mice with manipulated S1P signaling pathways.

### 4.3. Complex Action of S1P in Vascular Tone Control and Hypertension

Several studies have revealed that S1P/S1PRs signaling promote both vasorelaxation via ECs and vasoconstriction by VSMCs, thus regulating blood pressure in a balanced way. This process involves not only store-operated calcium channels (SOCs) in VSMCs [86] but also endothelial K_Ca_2.3 and K_Ca_3.1 channels in ECs. Blocking K_Ca_2.3 and K_Ca_3.1 channels attenuated the vasodilation response caused by acute S1P stimulation of the mesenteric arteries [87]. Aldosterone has been identified as an important factor in obesity-associated hypertension. It is suggested that S1P, the selective S1PR1 agonist SEW2871, and the selective S1PR2 antagonist JTE013 increase aldosterone secretion in NCI H295R cells [88]. Treatment with S1PR1 antagonists W146 or FTY720 and S1PR1/3 antagonist VPbib2319 decreased baseline and/or S1P-stimulated aldosterone release [88]. The administration of ApoM-Fc, a soluble carrier of S1P, reduced blood pressure in hypertensive mice by activating S1PR1 and S1PR3 [89]. Interestingly, it has been reported that S1P mediates endothelial-dependent vasodilation by NO at low concentrations (nanomolar range), whereas at high concentrations (1–100 μM, such as those reached in the presence of thrombin) it initiates vasoconstriction [90]. The role of S1P in blood pressure homeostasis still needs further investigation to clarify the contribution of different downstream pathways.

### 4.4. S1P Reduces Myocardial Ischemia and Reinfusion Injury

S1P is an important endogenous cardioprotective agent in myocardial ischemia and reinfusion (I/R) injury [91]. S1P may be the key constituent responsible for the cardioprotective effect of HDL, with actions in both the preconditioning and postconditioning processes [92]. It also regulates endothelial dysfunction and immune cell behavior to control vascular permeability and immune cell immersion at the site of I/R injury [93]. S1P treatment reduced the area of infarction in cardiac I/R via S1PR3-RhoA signaling, and the protective effect disappeared in S1PR3-deficient mice and mice receiving S1PR inhibitors [94]. In an isolated rat heart model of acute heart failure, the effect of S1P was associated with increased phosphorylation of STAT3 [95]. Although these reports have all confirmed the role of S1P in I/R, further studies are still required to illustrate more detailed mechanisms.

### 4.5. Cardioprotective Effects of S1P in Myocardial Infarction, Fibrosis and Heart Failure

Myocardial infarction (MI) is caused by acute coronary occlusion and leads to myocardial injury in the corresponding area of the blood supply [96]. After MI, the myocardium is remodeled and often proceeds to heart failure, characterized by cardiomyocyte hypertrophy changes in the extracellular matrix (ECM) and apoptosis. The remodeling of ECM with excessive collagen accumulation is a pathological process of myocardial fibrosis seen in ischemic and nonischemic cardiomyopathies [97]. Studies have shown that S1P/S1PR1 mediates the involvement of exosomes in cardiac protection after MI through negative regulation of inflammation, inhibition of myocardial apoptosis, and fibrosis [98,99]. S1P pretreatment significantly enhanced adipose tissue-derived mesenchymal stem cell (AT-MSCs) migration and antiapoptotic effects to improve MI. The mechanism suggested that S1P/S1PR2 promotes migration by activating ERK1/2-MMP-9 and inhibits apoptosis by activating AKT in AT-MSCs [100]. They observed that a loss of S1PR2 and S1PR3 increased the infarct area, and preischemic intravascular administration of S1P worsened the recovery of cardiac function and increased the infarct area in both wild-type and S1PR3-KO hearts, although S1PR3 deficiency attenuated coronary perfusion [101]. In addition, they found that the upregulation of SphK1 and S1PR1 caused an increase in cardiac S1P after myocardial infarction, while the SphK1 inhibitor inhibited S1P and improved cardiac insufficiency. Another study confirmed that the SphK1/S1P/S1PR1 signaling pathway activates the proinflammatory response after myocardial infarction [102].

Fibroblasts are an important cell type in the heart and appear to be an important source of endogenous S1P in the heart with higher levels of SphK activity than cardiomyocytes [103]. Studies have shown that S1P plays an important role in the regulation of cardiac fibroblast proliferation, migration, differentiation, and survival [104]. S1PR2 mainly mediates fibroblast transformation and collagen production by activating small GTPases and Rho through Gα_12/13_ [105]. The Rho kinase inhibitor (Y-27632) suppressed cardiomyocyte hypertrophy and interstitial fibrosis in mice postmyocardial infarction (MI) [106]. Rho kinase knockout mice exhibited a dramatic decrease in cardiac fibrosis, confirming the function of Rho in regulating fibroblast transformation [107]. Fibroblasts transform into myofibroblasts and produce extracellular matrix (ECM) in response to injury, where, a key factor, the transforming growth factor-β (TGF-β) is involved [108]. It is also reported that TGF-β stimulates production of S1P, via increased SphK1 activity and expression [103]. Moreover, S1PR1 participates in the regulation of cardiac fibrosis [101]. A recent study demonstrated that S1P upregulates the expression of cyclooxygenase-2 (COX-2), activates NF-κB via S1PRs /PKCα/MAPKs cascade, and increases prostaglandin E_2_ (PGE_2_) generation, which eventually results in apoptosis of human cardiac fibroblasts [109]. 

During myocardial remodeling, the S1P/S1PR1 pathway promotes reparative macrophage proliferation [110] and tightly controls macrophage trafficking via lymphatic vessels [111] in injured hearts, thus ameliorating post-MI adverse cardiac remodeling. Chronic activation of SphK1-S1P signaling exhibits cardioprotective effects in pathological cardiac remodeling through ROS mediated by S1PR3 in transgenic mice [112]. S1PR3 is highly expressed in fibroblasts. Keller et al. [113] demonstrated that FTY720 induced transformation of fibroblasts into myofibroblasts and mediated myofibroblast differentiation through S1PR3/Smad3 signaling and thus induces cardiac fibrosis. The adverse fibrotic effect of FTY720 was abolished in S1PR3 knockout mice. However, Ohkura et al. [99] generated transgenic mice with cardiac fibroblasts overexpressing S1PR1, and the mouse hearts developed bi-ventricular hypertrophy and diffuse interstitial fibrosis without hemodynamic stress. Sphingolipids are significantly associated with the development and progression of heart failure. Plasma S1P and sphingomyelins levels are significantly negatively correlated with left ventricular ejection fraction and severity of dyspnea [114]. In general, S1P plays a protective role against acute heart failure and promotes cardiac remodeling during chronic heart failure.

## 5. Conclusions

S1P is a biologically active lipid molecule involved in the regulation of various cardiovascular functions. The differential expression of its three receptors (S1PR1-3) in different cell types, along with the diversity of G proteins they couple, determine the downstream effects of S1P. S1P synthase, transporters, and metabolic enzymes also affect cardiovascular function by the dynamic control of S1P levels. Due to the complex regulatory network, S1P signaling shows multifaceted and sometimes opposite effects on the cardiovascular system. Overall, S1P is a cardioprotective factor against several types of myocardial injury and can play either a beneficial or harmful role in vascular diseases (Table 1). In recent years, as the structures of S1PRs have been revealed [32,37], more agonists or antagonists of S1PRs with higher isoform selectivity can be rationally designed. This will hopefully provide more tools for further investigation the signaling mechanisms of S1P in cardiovascular diseases. In addition, more clinical studies with currently approved S1PR modulators are needed to explore potential therapies for various cardiovascular diseases. 

## Figures and Tables

**Table 1:** The roles of S1PRs in cell types associated with cardiovascular diseases.

Cell Type	Function	Role of S1PRs	Associated Diseases
Endothelial cell	Barrier maintenance, angiogenesis, lymphocyte trafficking, vascular tone, and inflammation	S1PR1: protect or rescue endothelial barrier function; promote endothelium-dependent vasorelaxation	Atherosclerosis, angiogenesis, and hypertension
		S1PR2: disrupt adhesion junctions; increase paracellular permeability	
		S1PR3: mediate cell proliferation and vascular permeability; a potential biomarker in acute lung injury (ALI); promote endothelium-dependent vasorelaxation	
Vascular smooth muscle cell	Angiogenesis, inflammation, and vascular tone	S1PR1-3: regulate VSMC proliferation, migration and VSMC-dependent vascular tone	
		S1PR2/3: mediate vasoconstriction and anti-inflammation	
Renal medullary epithelial cell	Water and sodium homeostasis	S1PR1: increase urine flow and sodium excretion; regulate blood pressure through effects on blood volume	
Cardiomyocyte	Affect cardiac muscle contraction, cardiomyocyte viability, and heart rhythm	S1PR1: produce the negative inotropic effects by inhibiting L-type calcium channel current, activating inwardly rectifying K^+^ current and shortening action potential duration (APD)	Myocardial ischemia and reinfusion (I/R) injury, cardiac hypertrophy, myocardial infarction, fibrosis and heart failure
		S1PR2/3: lead to negative inotropic response by inhibiting isoproterenol-stimulated cAMP accumulation	
Fibroblast	Regulation of cardiac fibroblast proliferation, migration, differentiation, and survival	S1PR1: regulate of cardiac fibrosis	
		S1PR2: mediate fibroblast transformation and collagen production	
		S1PR3: mediate myofibroblast differentiation	

## Data Availability

Not applicable.
